# Alcoholic Lotion in Phthisis Pulmonalis

**Published:** 1844-09

**Authors:** Marshall Hall


*On the use of the Alcoholic Lotion in Phthisis Pulmonalis.* By Marshall Hall, M.D., F.R.S., &c.—So many persons affected by incipient phthisis, marked by dulness of sound on percussion, and no doubtful pectoriloquy under the clavicle, hæmoptysis, and disposition to chills, heats, and early morning perspirations, &c., have been benefitted and restored to apparent health by the remedy or remedies, which I am about to mention, that I cannot but think they possess great efficacy.

The first and the principal of the remedies is an alcoholic lotion constantly applied by means of six folds of linen over and across the upper lobes of the lungs.

One part of pure alcohol is mixed with three parts of water. It is applied tepid at first, afterwards of the temperature of the atmosphere. It is applied in *small* quantity at a time, every *five* minutes, so that the application may always consist of alcohol and water. (If applied in larger quantity and less frequently, the alcohol would evaporate, and water alone would be left, and this would be the source of a feeling of discomfort instead of the feeling of glow which the alcohol induces.) The application is easily made; a piece of soft linen, of the size of a very *large* sheet of letter-paper, being folded in the usual manner, is then folded twice more, in lines parallel with the first, so that the whole consists of six folds. These are stretched, applied across the upper part of the thorax just below the clavicles, and fastened to the shoulder-straps, or other part of the dress, which latter is to be arranged so as to be readily opened and closed. A sponge, the size of a walnut, is then filled with the lotion, and pressed upon the linen along its whole course, the dress being opened for this purpose and immediately closed.

This operation need not occupy five seconds. It should be repeated, as I have stated, every five minutes. The application of the lotion should be incessant during the day and all waking hours, the dress being light, or even entirely removed, so as to allow of free and rapid evaporation. It is suspended during the night.

It is by no means my wish to laud this remedy beyond its just value; but I have no hesitation in asserting that it possesses a power in checking the progress of the deposition and softening of tubercle in the lungs, beyond any other which I have tried. And the number of patients who have recovered from incipient phthisis under its use, and who, after many years, are still living, and in apparent health, induces me to express myself in strong terms in regard to its extreme value.

One patient, who consulted me fifteen years ago, had dulness on percussion, and pectoriloquy, and every other sign of incipient phthisis. He applied, and long wore, the alcoholic lotion, called it his "breast-plate," and is now a professor of ——— College.

A lady, about thirty years of age, became affected with hæmoptysis. She was enjoined the alcoholic lotion. It is fourteen years since it was first applied, and it is continued, or renewed, if ever suspended, to this day.

I saw a young lady two years ago, one of a most consumptive family, affected with hæmoptysis, with every threatening sign and symptom of incipient phthisis. I prescribed the alcoholic lotion, and the cough and hæmoptysis were removed, and every fear dispelled. It had already been proposed that this young lady should take a voyage to Madeira. She did so, continuing the lotion, and returned in apparent good health.

Three months ago a young lady was brought to me, having a recurrence of hæmoptysis. There were pectoriloquy and dulness under the right clavicle, cough, loss of color, and of flesh. The alcoholic lotion was applied. The hæmoptysis and cough ceased. The patient went to Hastings, and every account which I have received has been one of improved health.

I give these cases as examples. I do not imagine that the alcoholic lotion does more than *check* the morbid process. But—"Est quoddam prodire, tenus si non datur ultra."

In what the morbid processes of the deposition and the softening of tubercle consist, I believe we do not know; but if these processes be really checked by the application of the alcoholic lotion, we have a *practical* fact which must excite the deepest interest. Some degree of this influence, in incipient cases, is, I believe, exerted by this remedy.

None of the remedies ever yet proposed for phthisis has maintained the character first given to it. The encomiums bestowed upon them were always beyond the truth; I would, therefore, carefully guard against such an event in the present instance; and I would beg to be understood as stating only the fact that I have witnessed many, very many, cases of incipient phthisis checked by the strenuous application of the alcoholic lotion, and the patients restored to *apparent* health, these cases having been proved to be phthisis by the presence of the physical signs, as well as the morbid symptoms of this dire disease.

I would also guard my readers against trusting to this remedy as a sort of *cure* for phthisis. I think it the most important remedy in this disease which we possess; but I would by no means neglect *any* of the other well-known *aids* in the treatment of phthisis. Of these, changes of air; free exposure to the sea-breezes; a sea voyage; a mild climate; a chalky soil; a locality screened from the north-east winds; gentle exercises, especially on horseback; a meat diet, with a little of Bass's ale, perhaps, but otherwise without stimulus; the system of sponging with the sea water, or salt and water, or vinegar and water; light clothing, with flannel next the skin, &c., the plan recommended by the late Dr. Stewart, &c., constitute the additional remedies to be adopted in the treatment of phthisis."—*London Lancet.*

